# Medical Laboratory Science Education in Australia: An Academic Review

**DOI:** 10.1007/s40670-024-02057-1

**Published:** 2024-04-30

**Authors:** Rebecca Donkin, Rebecca Gusset

**Affiliations:** 1 School of Medicine and Dentistry, Griffith University, Sunshine Coast Health Institute, 6 Doherty St, Birtinya, Qld 4575 Australia; 2 School of Health, University of the Sunshine Coast, 90 Sippy Downs Drive, Sippy Downs, Qld 4556 Australia

**Keywords:** Medical Laboratory Science, Academic, Education, Graduates

## Abstract

Medical Laboratory Scientists contribute to pathology organizations to provide medical testing for the diagnosis, treatment, and prevention of disease. To meet patient medical testing demands in Australia, an employment projection of moderate growth by 2026 in Medical Laboratory Scientists is predicted. This requires an experienced academic workforce that is competent in Medical Laboratory Science (MLS) teaching supported by MLS research to graduate skilled MLS students to fill this void. However, there is little known about the academics that teach undergraduate MLS and whether there is a shortage of experienced educators and graduates. A mixed-method descriptive cross-sectional study design was used to identify 125 MLS academics to recruit and collect quantitative and qualitative survey data from 2019 to 2021. Over half of the survey respondents had never worked as a Medical Laboratory Scientist, and less than a third had an undergraduate degree in MLS. The breadth and depth of academic teaching and research interest were wide and covered both MLS and non-MLS themes. The retention of MLS academics remained stable. There was a meagre growth in new appointments over 3 years which was likely impacted by the COVID-19 pandemic which also impacted student enrolment and graduate data. It is unclear from these results if the 2026 predicted growth will be achievable.

## Background

The overarching role of a Medical Laboratory Scientist employed in a medical pathology service is to provide competent medical testing within a quality control system that determines the cause and nature of diseases [1]. In Australia, Medical Laboratory Scientists contribute to this service in public and private pathology organizations and most meet university accreditation requirements by the Australian Institute of Medical and Clinical Scientists (AIMS). Tertiary undergraduate programs in Medical Laboratory Science (MLS) provide theoretical and practical knowledge with combined clinical practice learning in pathology organizations to produce graduates eligible to be employed as Medical Laboratory Scientists. In Australia, at the end of 2021, 12 undergraduate university programs were accredited by AIMS [2].

University education for MLS is mostly provided by academics who teach general science and health subjects such as anatomy, physiology, and foundations in medical science. Some academics also specialize in MLS subjects that require in-depth practical expertise such as clinical chemistry, hematology, transfusion science, medical microbiology, and histopathology and have a research interest in MLS [3]. However, not all academics that teach in MLS programs have a university qualification in MLS or have experience as a clinical Medical Laboratory Scientist in a pathology laboratory. Previous literature has reported the level of appointment and research track level of Australian MLS academics [3]; however, there is a paucity of literature that describes the demographics of MLS academics, their qualifications, and previous experience as a clinical Medical Laboratory Scientist.

A report by the Victorian Allied Health Workforce Research Program [4] investigated the Medical Laboratory Science (MLS) workforce in Victoria where most Australian Medical Laboratory Scientists are employed (32% of all employed in Australia) [5]. This study identified that the predominant first qualification to practice as a Medical Laboratory Scientist was a university directed MLS bachelor degree (64%, *n* = 265) followed by 5% (*n* = 19) entering the profession with a graduate entry MLS master’s degree and the remaining having a range of other post-graduate qualifications including graduate certificates (*n* = 21), graduate diplomas (*n* = 51), Masters degrees (management, research or other *n* = 41), professional doctorates (*n* = 8), and PhDs (*n* = 24) [4]. This preliminary data highlights the knowledge gap in understanding who has a university accredited MLS qualification or relevant experience as a Medical Laboratory Scientist and if they enter academia, what experience they have in teaching specialized courses required for MLS programs.

The shortage of MLS and Clinical Laboratory Scientists (CLS) has been well-documented in high income countries [6, 7], which suggests that studies in Australian education are timely. There is growing concern that this will become more critical with an aging population and a greater need for pathology services, as well as the loss of experienced scientists retiring from the baby boomer era [6]. The problem becomes twofold when also addressing how university programs recruit and retain faculty to teach MLS programs who are adequately qualified and experienced and maintain a MLS research agenda. It is likely a shortage of academics who have clinical experience and qualifications in MLS will impact the overall quality of university programs and ultimately the quality of the MLS industry.

To intentionally retain experienced MLS faculty, it is a requirement of Australian universities for academics to hold a higher degree qualification, have a research agenda, and meet the guidelines for the Higher Education Standards Framework 2015 [8]. It is not stipulated that academics must have MLS experience, but this may be desirable. The standards specify that “academic teaching staff must be qualified to at least one level of qualification (AQF level or equivalent) higher than the course of study being taught or have equivalent relevant academic or professional or practice-based experience and expertise” [9]. This means that to fulfill the AQF + 1 rule an academic teaching in a MLS bachelor’s degree must hold a qualification above a bachelor’s degree such as a Masters or PhD, which would require research as a priority to obtain this qualification. However, there is no current evidence that a research priority improves retention strategies or improves the efficacy of teaching in MLS.

Because research is linked to academia and level of appointment, a previous study [3] benchmarked the research track record of Australian MLS academics and provided insight into how research productivity informed the level of appointment of academics across their career pathway. This study revealed how the employee’s institution (metropolitan versus regional) and research interest appeared to influence publication number, h-index, and citation scores. A follow-up study that incorporates the potential effect of the COVID-19 pandemic has not been conducted to understand whether attrition or change in the level of appointment occurred during the pandemic and if research productivity was influenced during this period. Although benchmarking across and within universities is a tool to evaluate the performance of a program and maintain accreditation requirements, this is rarely published. Moreover, there is a lack of literature that reports student outcomes and depicts accurate data on the number of students and graduates of MLS programs and employment outcomes for graduates of Australian MLS Programs [10].

From other allied health disciplines in Australia [11, 12], we know that academic role modeling builds student confidence and promotes the development of a student’s professional identity. Academic role modeling during teaching requires a level of professional experience in the field (in this case MLS) to draw upon. However, in MLS, there is limited research that accurately identifies the characteristics of an MLS academic. We hypothesize that MLS programs mostly employ faculty members without occupation-specific experience and a research track record that may not benefit graduate outcomes for students.

The specific research questions this study explored were the following:

1. What are the academic qualifications, teaching, research, and clinical MLS experience of MLS academics in Australia from 2019 to 2021?
2. What is the comparative data of the research track record and level of appointment of Australian Medical Laboratory Science academics from 2019 to 2021?
3. What is the number of students enrolled and graduated from MLS programs in Australia from 2019 to 2021?

We anticipate knowledge attained from this study will inform the tertiary sector on data of academics teaching in MLS programs in Australia and provide further data on whether a longitudinal change has or has not occurred.

## Methods

A mixed-method descriptive cross-sectional study design was used to first identify MLS academics from publicly available website information from Australian universities that had an undergraduate program of study in Medical Laboratory Science, Medical Science (Pathology), Science (Laboratory Medicine), or Laboratory Medicine relevant to pathology and met accreditation requirements by the Australian Institute of Medical and Clinical Scientists (AIMS). Secondly, purposeful recruitment of MLS academics and MLS program coordinators from AIMS accredited programs in Australia was done to collect quantitative and qualitative data through an online survey questionnaire.

A previous study [3] had already collated a list of academics in MLS programs in Australia, and this list was updated in 2021 using the same methods prior to recruitment. This included updating a bibliographic analysis of Australian MLS faculty websites and corresponding Scopus citation database profiles. A description of the current research track record and relationship with academic appointment level and institutional characteristics were explored directly from the database profiles. Research data was subsequently correlated by the Scopus citation database which included collecting accurate data on the number of publications, number of citations, number of co-authors, and h-index for each academic. Quantitative data and frequencies were analyzed using IBM SPSS version 26 to extrapolate data by research track record, qualification, gender, and academic appointment level.

From this list of academics collated from 2019 to 2021 (*n* = 125), an invitation was sent to participate in a survey questionnaire to obtain qualitative data to provide further information that could not be collected from publicly available quantitative data. Written informed consent, built into the online survey (Qualtrics, 2019 Provo, Utah, USA), was obtained when participants completed the online survey questionnaire. Participation in this study was completely voluntary, and participants could withdraw at any time. All methods were carried out in accordance with relevant guidelines and regulations, and this study received ethical approval from the Human Ethics Committee, University of the Sunshine Coast, A211550.

The survey collected academic characteristics such as the university academic program and level of appointment (Associate Lecturer equivalent to Instructor, Lecturer, Senior Lecturer equivalent to Assistant Professor, Associate Professor, and Professor) to correlate the quantitative data. Along with previous education qualifications or previous work experience related to MLS, and years of experience, for example, the discipline area and years spent teaching or working as a MLS Scientist in a pathology laboratory, which could not necessarily be captured through database profiles. Thematic analysis [13] was used to extrapolate a theme or keyword from open-ended questions in relation to MLS research and teaching strengths and weaknesses. Data on student graduate and employment outcomes were collated through the university MLS program coordinator where available.

For descriptive statistics, the relative and absolute frequencies were calculated, as well as the mean (SD), frequencies, and percentages. Possible associations between academic and level of appointment characteristics were analyzed using appropriate correlational and bivariate tests for significance.

## Results

### Survey Questionnaire

Academic staff (*n* = 31) from 10 AIMS accredited Australian universities completed the survey, (response rate 25%). The majority of responders were employed as a Lecturer (*n* = 11, 35.5%) followed by Senior Lecturer (equivalent to an Assistant Professor, *n* = 10, 32%), Associate Professor (*n* = 6, 19%), and Professor (*n* = 3, 10%), and one as a Program Director (*n* = 1, 3%). Academics had taught at the university level for 1–20 + years with most completing between 11 and 20 years of service (*n* = 12, 39%).

Prior undergraduate degrees in MLS were obtained by only nine (29%) of the 31 academics and were not necessarily obtained from AIMS accredited universities. The majority of academics (*n* = 28, 90%) were awarded a PhD with the remaining academics holding a Masters (*n* = 3, 10%). Over half of the respondents (*n* = 17, 55%) had no work experience as a Medical Laboratory Scientist or Technician in a clinical pathology laboratory. Of those academics (*N* = 14, 42%) that had work experience in a clinical pathology laboratory, nine had worked more than 10 years, and five had worked 10 or fewer years in a wide field of MLS disciplines or worked multi-disciplinary. The most common work experience was in the discipline of Hematology (*n* = 7, 15%), followed by Transfusion and Clinical Chemistry (both *n* = 5, 11%), and then Clinical Microbiology (*n* = 3, 6%). The least common areas of work experience were in quality control (*n* = 2, 4%) and Anatomical Pathology/Cytology and Cytogenetics (*n* = 1, 2%). Only one academic was employed in an AIMS accredited program and was currently working as a Medical Laboratory Scientist.

### Academic Teaching

Academic respondents accumulatively taught 100 university subjects including basic science subjects such as Biochemistry, Physiology, and Pathophysiology as well as subjects specific to MLS programs including Pathology, Microbiology, Medical Biochemistry, Hematology, and Transfusion. Other subjects that were not relevant to an undergraduate MLS program were in non-MLS programs such as Medicine and Exercise Physiology.

**Fig. 1:**
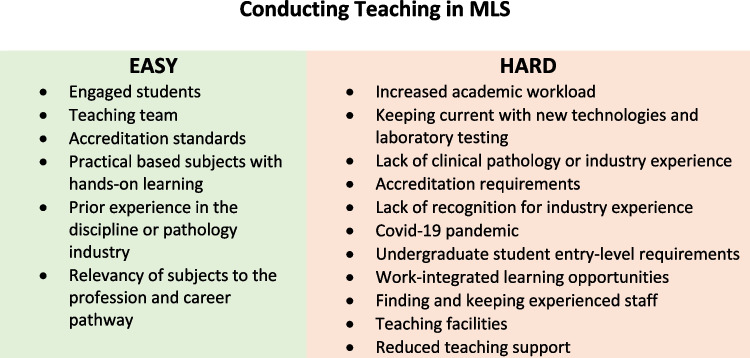
Factors that make it easy or hard to teach MLS

Academics were asked to reflect on factors that make it easy and difficult (harder) to teach MLS subjects. The thematic analysis provided six themes for ease of teaching and 11 themes for difficulty in teaching MLS. Figure 1 illustrates the key themes that make it easy and hard to teach MLS subjects reflected by the respondents which are alluded to further in the “Discussion” section.

### Benchmark of Research Track Record by Academic Appointment Level

Of the 31 academic respondents, the breadth of research interest was wide with both MLS and non-MLS areas of interest including cancer biochemistry, endocrinology, nutrition, environmental science, metabolomics, neurology, medical technology, simulation, student support and retention, and government policy. However, not all academics have published in their field of research.

The results from the online survey responses were collated to identify the major research interest that was comparable to the publicly available website information. The top six major research interests by the survey were molecular biology, cardiovascular, genetics, physiology, coagulation, and biotechnology. The survey data was correlated with the website data and five minor discrepancies were identified from the academic’s website which reported an area of research interest, but their publication record was stronger in a subsequent area of interest. This could be attributed to academics not updating their website details or that they had multiple areas of research interest that were not reflected by their publication record. The research track records of Australian MLS academics from 2021 and their corresponding Scopus citation database profiles were recorded as an accurate indexed publication recorded; further data is shown in Table 1.

**Table 1:** 2021 research track records of Australian Medical Laboratory Science academics by the level of appointment

		**Academic Level**					
		**Associate Lecturer (*N* = *2*)**	**Lecturer (*N* = *32*)**	**Senior Lecturer (*N* = *52*)**	**Associate Professor (*N* = *31*)**	**Professor (*N* = *8*)**	**All *(N* = *125)***
Number of publications	Range Median	0–13 7	0–65 8	2–128 35	9–117 59	53–233 102	0–233
Number of citations	Range Median	0–319 160	0–2251 170	1–3837 896	301–7429 1236	1860–11631 2154	0–11631
Number of co-authors	Range Median	0–31 16	0–1772 23	3–1130 68	20–3040 119	127–660 157	0–3040
*h*-index	Range Median	0–8 4	0–27 6	1–31 15	7–37 19	23–65 26	0–65

**Fig. 2:**
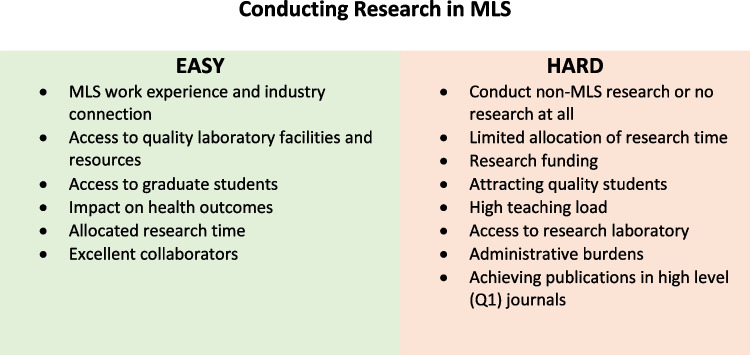
Factors that make it easy or hard to conduct research in MLS

For those academics that had published in an area of MLS research, they were asked to complete an open-ended question to reflect on factors that make it easy and difficult (harder) to conduct research in MLS areas. The thematic analysis provided six positive and eight negative themes (Fig. 2); further insight into these themes is provided in the “Discussion” section.

### MLS Student Enrolment and Graduate Outcomes

Of the 12 AIMS accredited universities invited to participate, seven responded with student graduate enrolment and outcome measures from 2019 to 2021, with one university introducing a new program in 2021. In 2019, a total of 1078 students were enrolled in MLS undergraduate degrees. In 2020, there was a meagre growth of 0.8% in enrolment to 1087 students. In 2021, a growth in enrolment of 12.1% occurred with 1219 students enrolled in a MLS program which may have been attributed to a new program commencing in 2021.

The number of students graduating from completed MLS undergraduate degrees from the seven responding AIMS accredited universities in 2019 was 247 (22.9% total enrolled), while in 2020 (with the start of the COVID-19 pandemic in Australia), only 189 (17.4% total enrolled) students graduated. Due to the timing of the survey at the end of 2021, not all universities had finalized their graduating numbers, and the predicted 2021 student graduation number was 211 (17.3% total enrolled).

### Retention and Attrition of MLS Academics During the COVID-19 Pandemic

From data collected in December 2019 from the publicly available website information, there were 124 academics (*n* = 68 male; *n* = 56 female) who had teaching and research positions in an undergraduate MLS program in Australia. In October 2021, there were 125 academics (*n* = 68 male; *n* = 57 female). There were 15 new academics and 14 academics who no longer were employed in a MLS university program.

In 2019, 79.2% (*n* = 99) held a PhD compared to 87.2% (*n* = 109) in 2021, which equates to an 8% increase in PhD qualifications over the 3 years. Holding a doctorate continued to strongly influence the number of publications in 2021 (median = 2 publications for those without a doctorate and 38 for those with a doctorate). The level of appointment was also reflected by an increase in promotion or new appointments from 2019 to 2021 from Lecturer to Senior Lecturer (*n* = 6) and Senior Lecturer to Associate Professor (*n* = 11) which was statistically significant (*p* < 0.001). The academic level of Senior Lecturer (equivalent to Assistant Professor) remained the predominant level in both 2019 (36.8%) and 2021 (41.6%); further details are provided in Table 2.

**Table 2:** Level of appointment by gender

**Level of appointment**	**2019 Total** ***n*** **(%)**	**Male** **(*n*)**	**Female (*n*)**	**2021 Total** ***n*** **(%)**	**Male** **(*n*)**	**Female (*n*)**	**Total change**
Associate Lecturer	2 (1.6%)	0	2	2 (1.6%)	1	1	0%
Lecturer	34 (27.2%)	18	16	32 (25.6%)	14	17	−1.6%
Senior Lecturer	46 (36.8%)	26	20	52 (41.6%)	31	21	+4.8%
Associate Professor	20 (16%)	11	9	31 (24.8%)	17	13	+8.8%
Professor	8 (6.4%)	5	3	8 (6.4%)	4	4	0%

**Fig. 3:**
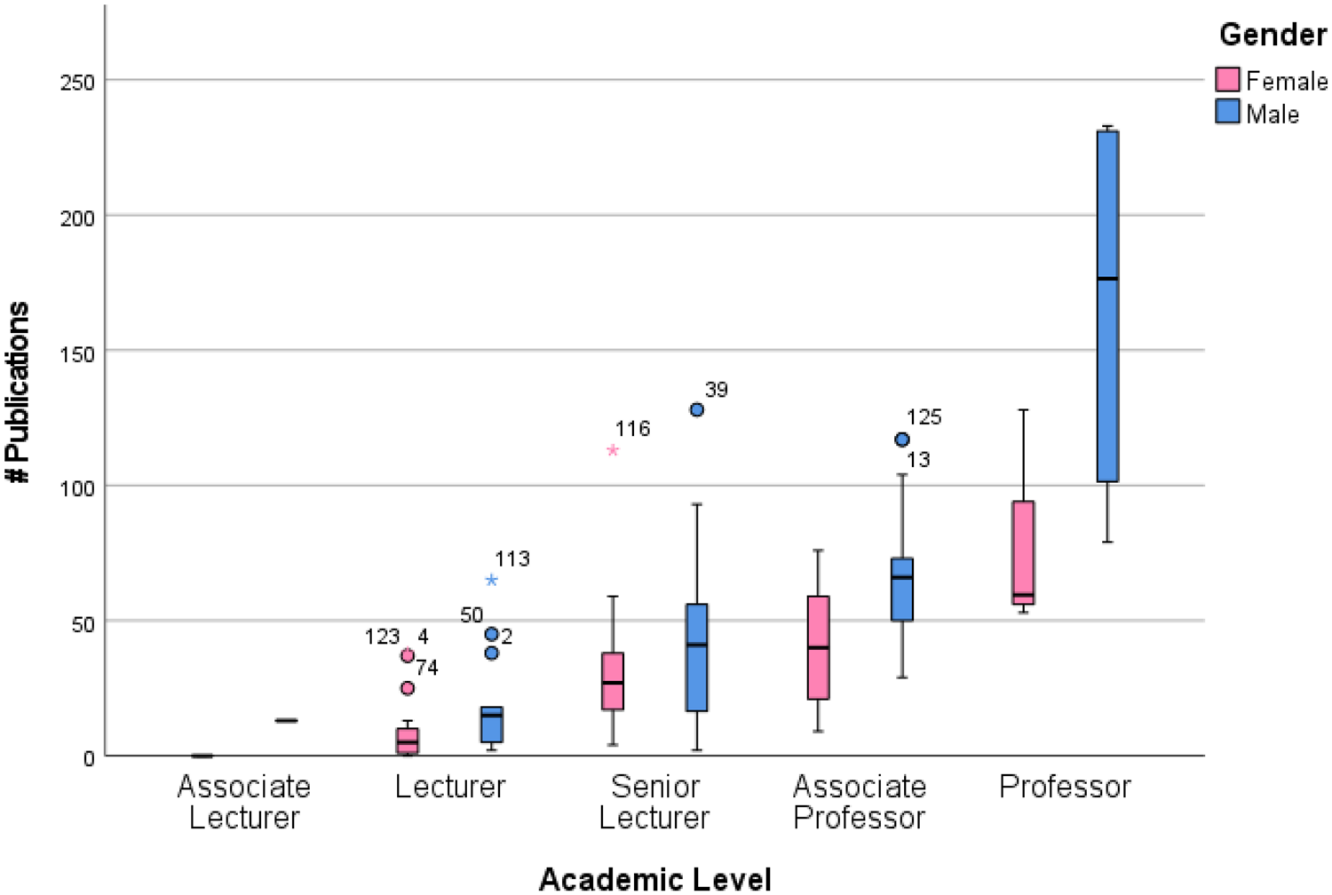
The number of publications in 2021 against academic level by gender

The proportion of male academics (54.4%) employed in a MLS program was higher than females (45.6%) in 2021, and the number of publications by gender was also reflected by this. The average publication frequency in 2021 was higher in males compared to females across all academic levels; however, gender was not an explanatory variable on the level of appointment (Fig. 3). Backwards linear regression was conducted to ascertain the impact of research track record and other explanatory variables (e.g., gender, number of publications, number of citations, number of co-authors, and h-index) on the level of appointment. A significant model (*p* < 0.001, *R*^2^ = 0.101) was found with only one explanatory variable, the number of publications (*B* = 0.004), and a constant 0.427. Confirmatory analysis of this model was conducted using ordinal regression, which resulted in a similar model (*p* < 0.001, *R*^2^ = 0.398) and the same explanatory variable.

Interestingly given the timing of the COVID-19 pandemic in 2020, the mean number of total publications over an academic career, statistically increased in 2021 (40.68 + / − 39.4) and was significantly higher than in 2019 (35.53 + / − 34.37, *p* < 0.001). This suggests an appreciable number of publications were produced in 2020, despite the pandemic, and could be explained by the lockdowns affording academics more time to dedicate to writing and publishing manuscripts. This finding is further deliberated in the “Discussion” section.

## Discussion

Data from this study explored three research questions. The first question studied the academic qualifications, teaching, research, and clinical MLS experience of MLS academics in Australia, and the second reflected on the comparative data of the research track record and level of appointment of Australian MLS academics from 2019 to 2021. Thirdly MLS student enrolment and graduate outcomes were collected.

### Academic Qualifications, Teaching, Research, and Clinical MLS Experience of MLS Academics

Interestingly, there was only a meagre growth in academic appointments from 2019 to 2021 which may have been impacted by the COVID-19 pandemic. Encouragingly more academics held a PhD and the level of appointment increased, reflected by promotions or new appointments with Senior Lecturer (equivalent to Assistant Professor) remaining at the predominant level in both 2019 and 2021. Not surprisingly, the level of appointment could be correlated to the research track record, with the number of publications as a significant explanatory variable. This highlights the necessity of a strong publication record for an academic appointment and the need for ongoing research in both academic and clinical research in pathology [3].

The breadth and depth of research interest were varied, and thematic analysis of the respondent’s answers to MLS research did not necessarily include traditional MLS themes. Comments supporting this included “I do maintain an active research portfolio [but not research in Medical Laboratory Science] it is in clinical trials and cohort studies.” Academics commented that it was hard to conduct MLS research when they were from a non-MLS background as “MLS research is not my major area of expertise,” or they could not attract high quality MLS students because “there is a general lack of interest in MLS research careers.” Obtaining grant funding for MLS topics was limited, or research time was limited due to heavy teaching loads, the impact of the COVID-19 pandemic, or the employment profile if the academic held a “teaching only academic role.” Academics commented that when they could conduct research it was made easier by access to funding, facilities, engaged students/collaborators, and doing research outside of MLS themes. It was also important to have an academic profile that included research time, “I have 40% time allocated for research as part of my continuing academic position, an excellent laboratory, and previous training.” This is in line with other studies in health-related fields that show increased research productivity when supported financially and with protected time to conduct research [14].

However, MLS specific research or MLS work experience does not appear to be a significant factor in MLS academic appointments with over half of the respondents without work experience as a Medical Laboratory Scientist or Technician in a private or public clinical pathology laboratory.

Teaching during the COVID-19 pandemic was also interrupted and reflected by comments that made it hard to teach MLS due to a “reduction of face-to-face teaching” and interruption to work-integrated learning placements that postponed student graduations. This impact was felt worldwide with a disruption to in-person teaching and an immediate online pivot of programs that specifically affected practical-based programs that were modified or could not be taught online due to the nature of the bench work or hands-on learning required (including work integrated learning placements) to meet accreditation standards [15–18].

More astounding were the comments from MLS academics that were not reflected by the pandemic but on self-importance and lack of recognition from peers as reported as “seen as a lightweight in terms of research outcomes and my previous work [experience as a MLS Scientist] not recognized and seen as being less important than research experience.”

Not having clinical experience in a pathology laboratory was a major theme of not being able to teach MLS well, “I don’t have clinical or industry experience” or “the fact that I am a basic research scientist (molecular biologist) and have never worked in a clinical laboratory makes it more difficult to teach students in this clinical program.” Interestingly, although having MLS work experience but not holding relevant qualifications (AQF+1) could preclude employment. “I couldn’t employ MLS staff as the university had restrictions on employing sessional staff with only industry experience.”

Contrary to this, it was reported that having a passion for teaching practical skills, work experience in diagnostic pathology laboratories “bench work,” and a PhD resulted in a positive teaching experience in MLS. Respondents reported that they found “knowledge and experience in MLS has helped understand Clinical Biochemistry and its relevance in the real world so embedding this discipline into courses is relevant” and additionally “I worked for many years as an MLS, therefore, I know what is relevant, I am interested in the field and it is rewarding to see students learn and progress”; furthermore, “I have gained invaluable experience as a Senior Medical Scientist which provides me with the current industry collaborations to keep me up to date with current content. This enables me to transfer this knowledge to students in an undergraduate setting.”

Positive affirmations suggest that experience and passion for MLS by academics make MLS teaching and research easier and likely convey enthusiasm and motivation for students to pursue a career in MLS. The insightful factors that make it easy or hard to teach and conduct research in MLS have the potential to expand the impact of teaching on other healthcare professional education programs. There are likely similar themes in accredited programs surrounding teaching and research opportunities and prior work experience which impact positive or negative perceptions of an academic role.

### Comparative Data of the Research Track Record and Level of Appointment of Australian Medical Laboratory Science Academics from 2019 to 2021

The academic level of Senior Lecturer (equivalent to Assistant Professor) remained the predominant appointed level in both 2019 and 2021, with an increase of six promotions or new appointments from 2019 to 2021 from Lecturer to Senior Lecturer. Research track record continues to be an important factor in the level of appointment. Consistent with previous research [3, 19], the number of publications was skewed toward the lower end relative to a lower appointment level and is likely attributed to less time in the profession. Interestingly, the mean number of total publications increased in 2021 and was significantly higher than in 2019 which was not consistent with other health academic programs during the COVID-19 pandemic [20]. Initially, lockdowns, restrictions, and funding to conduct research disrupted activities, and it was reported that the COVID-19 pandemic was associated with an 18% decrease in the production of non-COVID-19 research in health-related fields; however, publications in COVID-19 health-related articles increased substantially [21]. While it was beyond the scope of this study to assess each individual publication by the level of appointment, it was promising that research in MLS continued during the pandemic, which was possibly attributed to an increase in acceptance of COVID-19 publications, given the field of research in MLS.

However, an increase in research and subsequent publications during the COVID-19 pandemic did not affect all scientists equally. It has been reported that this was not the case for female scientists, [22] especially those conducting bench work in MLS and especially scientists with young children who were unable to devote time to research due to caring duties noted a substantial decline [23]. Of note, in MLS, the average publication frequency in 2019 and 2021 was higher in males compared to females across all academic levels; however, gender was not an explanatory variable on the level of appointment.

### Student Enrolment and Graduates in MLS

Of the 12 AIMS accredited universities invited to participate, seven responded with student graduate enrolment and outcome measures which reported a meagre growth in enrolment numbers and a decline in graduates. These outcomes were likely impacted by the number of students unable to complete their required clinical placements during the COVID-19 pandemic due to the on-site restrictions, which subsequently resulted in delayed graduations or increased attrition. This had a knock-on effect on the employment of MLS graduates to MLS Scientist positions during this time.

In Australia, in 2019, there were over 28,000 employed MLS Scientists. At the beginning of the pandemic in 2020, this dropped to less than 23,000 (similar to employment data in 2016) [5]. While employment figures have risen and are expected to grow moderately to reach 28,400 by 2026 in Australia [24], there will be demands on tertiary educators to graduate MLS students to meet this gap.

In Australia, Victoria continues to have the highest employment rate of MLS employees with almost a third (31.7%) employed as MLS Scientists. Around 82% of Medical Laboratory Scientists live in capital cities, and Victoria has a large share of employment relative to its population size, with Melbourne, the capital city of Victoria, with the largest share of employed MLS Scientists [5]. Interestingly, there is only one AIMS accredited MLS program in Victoria, the Bachelor of Biomedical Science (Laboratory Medicine), at the Royal Melbourne Institute of Technology (RMIT) that produces MLS graduates.

### Limitations

The authors are mindful that this research was conducted during the COVID-19 pandemic and data collected at this time may not be representative of actual or predicted data pre- or post-pandemic for all MLS university programs. The response rate to the online questionnaire was 25% (*n* = 31) and may not reflect all attitudes and experiences from the 125 identified MLS academics. Only seven of the 12 AIMS accredited universities responded with enrolment and graduate data, and results were reported on only data received in this study. However, university website information and Scopus research data did provide a wealth of information even without qualitative insight from MLS academics. Further investigation of the type of research publications would provide insight into the depth and diversity of research published in the MLS field and whether research productivity was influenced by COVID-19-specific publications.

## Conclusion

Over the period from November 2021 to November 2026, the number of Medical Laboratory Scientists is expected to grow moderately and is likely to reach 28,400 by 2026 in Australia [24]. To meet this demand, tertiary educators will be required to graduate skilled MLS students to fill this void. This requires an experienced academic workforce that is competent in MLS teaching supported by MLS research to deliver adequate outcomes.

The COVID-19 pandemic had a significant impact on Medical Laboratory Scientist employment in 2020, and a sharp decline was also experienced in the number of graduating students in 2020 which was likely attributed to hindered or delayed clinical placement learning to complete the requirements of the program.

At the university level, there appears to be a gap between academic characteristics and MLS experience to best advocate for AIMS accredited degrees and pathology employment. The majority of academics with specific MLS teaching focus do not correlate with a research focus, nor does prior experience as a MLS Scientist appear to influence subjects taught, with over 100 different subjects taught by the 31 respondents. It is unclear whether an academic with or without MLS qualifications or research experience better prepares students for employment in pathology. However, the research track record exemplified by the number of publications is a significant positive variable in the level of MLS academic appointment.

These findings are of interest to all health accredited university programs aside from MLS because they examine the notion of the relevance of qualification, work experience in the field, and whether experience with or without research promotes effective educators. The implications of the data collected in this study could be considered in other health faculties to determine whether these are consistent findings across health academics and health professions education programs.

## Data Availability

The datasets used and/or analyzed during the current study are available from the corresponding author on reasonable request.
